# Is Drotrecogin alfa (activated) for adults with severe sepsis, cost-effective in routine clinical practice?

**DOI:** 10.1186/cc10468

**Published:** 2011-09-26

**Authors:** M Zia Sadique, Richard Grieve, David A Harrison, Brian H Cuthbertson, Kathryn M Rowan

**Affiliations:** 1 Department of Health Services Research and Policy, London School of Hygiene and Tropical Medicine, 15-17 Tavistock Place, London, WC1H 9SH, UK; 2 Intensive Care National Audit & Research Centre, Tavistock House, Tavistock Square, London, WC1H 9HR, UK; 3 Department of Critical Care Medicine, Sunnybrook Health Sciences Centre, Bayview Avenue, Toronto, ON, M4N 3M5, Canada

## Abstract

**Introduction:**

Previous cost-effectiveness analyses (CEA) reported that Drotrecogin alfa (DrotAA) is cost-effective based on a Phase III clinical trial (PROWESS). There is little evidence on whether DrotAA is cost-effective in routine clinical practice. We assessed whether DrotAA is cost-effective in routine practice for adult patients with severe sepsis and multiple organ systems failing.

**Methods:**

This CEA used data from a prospective cohort study that compared DrotAA versus no DrotAA (control) for severe sepsis patients with multiple organ systems failing admitted to critical care units in England, Wales, and Northern Ireland. The cohort study used case-mix and mortality data from a national audit, linked with a separate audit of DrotAA infusions. Re-admissions to critical care and corresponding mortality were recorded for four years. Patients receiving DrotAA (*n *= 1,076) were matched to controls (*n *= 1,650) with a propensity score (Pscore), and Genetic Matching (GenMatch). The CEA projected long-term survival to report lifetime incremental costs per quality-adjusted life year (QALY) overall, and for subgroups with two or three to five organ systems failing at baseline.

**Results:**

The incremental costs per QALY for DrotAA were £30,000 overall, and £16,000 for the subgroups with three to five organ systems failing. For patients with two organ systems failing, DrotAA resulted in an average loss of one QALY at an incremental cost of £15,000. When the subgroup with two organ systems was restricted to patients receiving DrotAA within 24 hours, DrotAA led to a gain of 1.2 QALYs at a cost per QALY of £11,000. The results were robust to other assumptions including the approach taken to projecting long-term outcomes.

**Conclusions:**

DrotAA is cost-effective in routine practice for severe sepsis patients with three to five organ systems failing. For patients with two organ systems failing, this study could not provide unequivocal evidence on the cost-effectiveness of DrotAA.

## Introduction

Severe sepsis is the most common cause of death for patients admitted to critical care [1-3]. Recent international studies suggest that the annual incidence of severe sepsis is 50 to 100 cases per population of 100,000 [4]. Approximately 80% of critical care admissions with severe sepsis have multiple organ systems failing, and the associated hospital mortality is around 50%. Severe sepsis is associated with substantial health-care costs; in the US, the annual costs are approximately $17 billion [1,5-9]. Severe sepsis survivors have a lower quality of life than the age- and sex-matched general population [10,11].

There is ongoing debate about the effectiveness of severe sepsis therapies, including corticosteroids, intensive insulin therapy, and Drotrecogin alfa (activated) (DrotAA) (Xigris^®^; Eli Lilly and Company, Indianapolis, IN, USA) (also known as a recombinant human activated protein C). In particular, although DrotAA has been evaluated in several large, multicenter randomized controlled trials (RCTs) [12,13], concerns remain about the therapy's effectiveness, both overall and for particular patient subgroups. In 2001, the PROWESS (Protein C Worldwide Evaluation in Severe Sepsis) trial reported that a 96-hour intravenous infusion (24 μg/kg per hour) of DrotAA versus placebo reduced absolute mortality at 28 days by 6.1% [13]. However, subgroup analysis of the PROWESS trial suggested a benefit solely for 'high risk' patients, and the original US Food and Drug Administration (FDA) license was limited to this subgroup; for 'low risk' patients, concerns about side-effects and lack of benefit meant that a follow-up study was requested [14,15]. The European Medical Evaluation Agency (EMEA) license for DrotAA was granted under 'exceptional circumstances', indicating that the efficacy data were limited and annual reassessment was required. Both the FDA and the EMEA licensed DrotAA for use in patients with severe sepsis at high risk of death but differed in their definition of baseline risk of death. High risk of death was defined by the US label as an APACHE II (Acute Physiology and Chronic Health Evaluation II) score of 25 or more and by the European Union label as the presence of multiple organ failure. The subsequent ADDRESS (Administration of Drotrecogin alfa (activated) in Early Stage Severe Sepsis) trial included solely 'low risk' patients but was stopped early for futility [12]. A separate trial, RESOLVE (REsearching severe Sepsis and Organ dysfunction in children: a gLobal perspectiVE), was initiated to investigate the efficacy and safety of DrotAA in children [16] but did not report any beneficial effect of DrotAA and was also stopped early. A new RCT, 'PROWESS Shock' [17], will provide additional evidence on the efficacy of DrotAA for high-risk patients, specifically those with septic shock, but does not include a cost-effectiveness analysis (CEA).

For DrotAA, the relatively high drug costs - the acquisition cost of a 96-hour infusion is around £5,900 (2010 prices) - and associated critical care stay and the tradeoff between potential benefit and risk mean that rigorous CEA is particularly important. Previous studies reported that DrotAA was cost-effective for adults with severe sepsis [18-23], but because the studies were based on PROWESS, these findings may not apply to routine clinical practice. General concerns with the RCTs for DrotAA are that they were tightly regulated, included a narrow range of patients and centers, applied restrictive treatment protocols, focused on short-term endpoints (28-day mortality), and did not collect cost data. These concerns make the RCTs unsuitable for assessing the cost-effectiveness of DrotAA in routine clinical practice. Furthermore, previous studies did not assess the cost-effectiveness of DrotAA according to the baseline risk of death or the timing of DrotAA administration. Recent work has suggested that DrotAA may be more effective if given within 24 hours of admission [24,25].

In the absence of appropriate RCTs, CEA may use observational data [26-28]. Recent observational studies have compared outcomes for severe sepsis patients receiving DrotAA with outcomes for matched controls [4,29-36]. However, none of these studies assessed cost-effectiveness. We report cost-effectiveness of DrotAA by using data from a prospective cohort study that audited outcomes following DrotAA in routine clinical practice in England, Wales, and Northern Ireland [29].

This paper aims to assess the cost-effectiveness of DrotAA in routine clinical practice versus control for adult patients with severe sepsis and multiple organ systems failing. The results are presented both for the overall group (two to five organ systems failing within 24 hours of admission to the critical care unit) and for high- or low-risk subgroups, defined according to the number of organ systems failing (two or three to five).

## Materials and methods

### Overview

By extending a previous prospective cohort study [29], this CEA compared DrotAA versus no DrotAA (control) for adult patients who had severe sepsis or multiple organ systems failing and who were admitted to critical care units in England, Wales, or Northern Ireland. In brief, the previous study used audit data from routine clinical practice and applied inclusion and exclusion criteria based on PROWESS [13]. Data for each severe sepsis patient, either receiving DrotAA or not (controls), were linked to case mix, resource use, and cost data from a national audit. Patients receiving DrotAA were matched to controls. Readmissions to critical care and mortality were recorded for a follow-up period of 4 years. The study follows method guidelines and extrapolates from the observed data to report cost-effectiveness over the patients' lifetime [37]. Details of the data sources, estimation of costs and outcomes, and analytical methods are given below.

### Data source and DrotAA patients

The effectiveness and costs for patients receiving DrotAA versus control were estimated with data from the Case Mix Programme (CMP) coordinated by the Intensive Care National Audit & Research Centre (ICNARC). The CMP is a national comparative outcome audit and includes 91% of the adult general (mixed medical and surgical) critical care units (including intensive care, combined intensive care, and high-dependency care units) in England, Wales, and Northern Ireland. Prospective data on case mix, resource use, and outcomes were collected on consecutive admissions to each participating critical care unit (see Harrison and colleagues [38] for details). Case mix data were recorded within the first 24 hours following unit admission and include age, physiological measures, medical history, surgical status, and reason for admission. The physiology data are used to calculate important prognostic measures for case mix adjustment or subgroup analysis; these measures include acute physiology score (ICNARC model), baseline predicted probability of death (ICNARC model), and the number and type of organ systems failing within 24 hours following admission on the basis of PROWESS definitions. CMP data have been assessed to be of high quality and highly representative of all UK critical care units [39]. Support for the collection and use of patient-identifiable data without consent was obtained under section 60 of the UK Health and Social Care Act of 2001 (approval number PIAG 2-10[f]/2005).

In December 2002, ICNARC conducted a large, multicenter audit on the use of DrotAA and subsequent outcome. Overall, 112 units (57% of those invited) actively participated; these units were representative of all those in the CMP. Each unit collected data on DrotAA use and adverse events for each admitted patient who received DrotAA at any time during their stay in the critical care unit. To provide CMP data on case mix, resource use, and outcomes, the DrotAA and CMP data were linked.

Patients who were admitted with severe sepsis and multi-organ systems failing or who developed severe sepsis and multi-organ systems failing during the first 24 hours in the critical care unit were identified by criteria derived from PROWESS [13]. Severe sepsis was defined as evidence of infection and three or more systemic inflammatory response syndrome criteria. Multi-organ systems failing was defined as two or more organ systems failing (cardiovascular, respiratory, renal, hematological, or metabolic) during the first 24 hours [29]. Each patient who met these criteria and received DrotAA at a participating unit during the recruitment period was included (*n *= 1,076).

### Controls

Controls were CMP database patients who were defined as having severe sepsis and multiple organ systems failing during the first 24 hours following admission to critical care, according to the above criteria, but did not receive DrotAA. Of the four potential control pools considered in the previous study [29], the control pool judged *a priori *to have the patients and units most similar to those receiving DrotAA was chosen. The control pool consisted of admissions contemporaneous to those who received DrotAA in those critical care units that went on to use DrotAA but who had not yet had their first use of DrotAA in that unit (*n *= 1,650).

### Costs

All hospital costs for the DrotAA and controls were considered for the index hospital admission and for readmissions to the original critical care unit over the course of a 4-year period. Resource use data were recorded and combined with unit costs.

### Resource use measurement

Resource use data were collected on the duration of DrotAA infusion, the length of stay (LOS) in critical care, and the total LOS in acute hospital. Information on the time at which DrotAA infusion was commenced was recorded. Complete data on the duration of DrotAA infusion were available for 90% of DrotAA cases; for the remainder, it was known that the infusion was interrupted; they were assumed to have the infusion for 48, rather than 96, hours. Information on readmissions to the original critical care unit for a 4-year period, following the index admission, was available from the CMP for each DrotAA and control. Details on the number of readmissions and subsequent critical care and acute hospital LOSs were extracted. It was assumed that, after 4 years, there were no further morbidity costs attributable to the initial episode of severe sepsis [40].

### Unit costs

Unit costs of DrotAA were taken from the National Institute for Health and Clinical Excellence (NICE) Health Technology Assessment (HTA) report [41]. For all admissions, each critical care bed-day was categorized according to the number of organ systems failing and costed with the corresponding cost per bed-day from the UK 'Payment by Results' database [42]. Costs per bed-day in hospital after discharge from critical care were taken from the literature [19]. All costs were adjusted to 2010-2011 price levels [43]. Resource use and unit costs were combined to report total costs per patient over the lifetime for DrotAA patients and for controls.

### Outcomes

The main outcome measure was the quality-adjusted life year (QALY) over the patient's lifetime. This measure required using data on mortality from the original critical care unit admission and ensuing hospital episode, and from any subsequent readmissions to that critical care unit, to project life years for each patient. These estimated life years were combined with estimates of health-related quality of life (HRQOL) [44] to project lifetime QALYs for each patient.

### Mortality

For the initial admission to critical care, information on vital status at acute hospital discharge and the date of death was taken from the CMP Database. For severe sepsis patients surviving the initial acute hospital episode, information on mortality following readmission to the same critical care unit, over 4 years, was used to reflect the excess risk of death in comparison with the general population [40,45]. For each patient whose death was recorded within those 4 years, the number of life years was then calculated directly (from the difference between date of death and date of initial admission). Patients who survived the initial acute hospital episode and all readmissions to the same critical care unit were assumed to have the same life expectancy, matched for age and gender, as the general population. There is evidence to suggest that severe sepsis survivors face a higher probability of death after the critical care episode in comparison with the general population (age- and gender-matched). However, there is no clear evidence of the duration of this excess mortality; the strongest evidence is in support of an excess mortality of up to 5 years, although some previous work has applied excess mortality for up to 25 years [46]. In our base case analysis, we follow previous studies [40,45,47] and take a conservative approach and apply the excess mortality for up to 4 years (see subsequent sensitivity analysis).

### Health-related quality of life

HRQOL for critical care survivors is lower than that for the general population after matching for age and gender [45,47,48]. We therefore down-weighted age- and gender-specific HRQOL weights from the general population [49]. We assumed, on the basis of the best available evidence from the literature [45], that HRQOL for critical care survivors was 80% that of the general population. We applied, on the basis of this long-term study, this adjustment factor for the 4 years following the initial admission, after which, HRQOL weights from the general population were applied (see subsequent sensitivity analysis, in which length of decrement of HRQOL is varied). Lifetime QALYs were reported by combining life years and HRQOL. Future QALYs and costs were discounted at the recommended rate of 3.5% [37].

### Analysis

#### Matching

Matched cohort analyses were performed; initially, this was done according to a propensity score (Pscore). The Pscore model was similar to that developed previously [29] and estimated the probability of receiving DrotAA with a logistic regression model that included both patient and critical care unit level baseline factors. Unit factors included were hospital type and number of critical care beds. Patient factors included were as follows: age, ICNARC model physiology score (IMscore), gender, number of organ systems failing, specific organ failures (cardiovascular, respiratory, renal, hematological, and metabolic acidosis), source of admission to critical care (via the emergency department, theater or recovery, ward, clinic or home), diagnostic category (ICNARC Coding Method) [50], and serious condition in the medical history [29]. Age and IMscore were defined as nonlinear terms fitted as smoothed functions by using restricted cubic splines [51]; other continuous measures were assumed to have linear relationships with treatment assignment.

We then applied Genetic Matching (GenMatch), which extends Pscore matching by using an automated search algorithm to choose the best matches, which are those that maximize the balance in the distribution of baseline characteristics between the treatment and control groups [28,52-55]. The explicit aim of GenMatch is to maximize balance according to statistics such as *t *tests or standardized differences in means. GenMatch matches on the Pscore but also matches on individual factors and can achieve better balance than matching on the Pscore alone [28,52-55]. A key advantage versus Pscore approaches is that GenMatch matches directly on any individual covariates judged to be potential confounders. Both methods matched one-to-one with replacement. So that average treatment effects could be estimated, each DrotAA case was matched to a control, and the remaining unmatched controls were each matched to a DrotAA case [56]. For both approaches, we reported balance on those variables previously judged to be important potential confounders [29]; these were age, gender, serious conditions in the medical history, acute physiology score (ICNARC model), predicted probability of acute hospital mortality (ICNARC model), proportion receiving mechanical ventilation, and number and types of organ systems failing during the first 24 hours in critical care. We reported covariate balance for the overall sample of DrotAA patients versus controls. Balance was measured with standardized mean difference; a difference of greater than 10% was taken to indicate meaningful imbalance [57,58]. Both matching methods were initially performed on the full sample of DrotAA patients and controls to report overall cost-effectiveness.

We categorized patients *a priori *into two subgroups according to the number of organ systems failing during the first 24 hours in critical care: two organ systems failing (low-risk subgroup) and three to five organ systems failing (high-risk subgroup). These particular subgroup definitions were adopted previously [29,41]. We matched DrotAA patients and controls with both matching methods, separately for each subgroup, and reported the resultant balance statistics at the subgroup level.

#### Statistical analysis

The aim of the statistical analysis was to report the incremental effectiveness, costs, and cost-effectiveness of DrotAA versus control. We reported average treatment effect for the overall group of patients (two or more organ systems failing at baseline) and for the *a priori*-defined subgroups of patients with two or with three to five organ systems failing. We reported the incremental effectiveness for DrotAA versus control as the odds ratio of acute hospital mortality at initial admission by applying logistic regression to the matched data. We compared mean costs and QALYs over the lifetime (DrotAA versus control). The CEA reported incremental cost-effectiveness ratios (ICERs) (costs per QALY) and incremental net benefits (INBs) [59] of DrotAA versus control. The INBs were calculated by valuing incremental QALYs according to the recommended level of willingness to pay for a QALY in the UK (£20,000 per QALY [37]) and subtracting from this the incremental costs.

Statistical uncertainty around the incremental results was considered by reporting 95% confidence intervals (CIs) by using the non-parametric bootstrap [60] on the matched data. One thousand bootstrap samples of the mean effectiveness and the mean costs were generated. The bootstrap recognized the correlation between costs and outcomes by bivariate re-sampling, and the re-sampling also stratified by treatment group. Incremental costs, incremental effects, and INB were calculated from the bootstrap sample. A cost-effectiveness acceptability curve [61] was constructed to report the probability that DrotAA was cost-effective at alternative levels of willingness to pay for a QALY gained.

#### Sensitivity analysis

The base case made the following assumptions that, while conservative, were judged to be potentially important: (a) GenMatch was the most appropriate matching method, (b) it was appropriate to include all DrotAA cases irrespective of the time infusion commenced, (c) DrotAA patients who did not have a complete duration of infusion and survived were assumed to have received 50% (48 hours) of the full infusion, (d) the excess mortality associated with severe sepsis was assumed for 4 years after critical care discharge, and (e) the decrement in quality of life for severe sepsis was applied for 4 years after hospital discharge. The sensitivity analyses tested whether the base case results were robust if the following alternative standpoints were taken:

##### Pscore matching

To test whether the results were sensitive to the matching method, cost-effectiveness estimates were reported after Pscore matching rather than GenMatch.

##### Timing of DrotAA infusion

This sensitivity analysis included only those DrotAA patients whose infusion was commenced within 24 hours of admission. This restricted sample was re-matched by using data from the original pool of controls. The main purpose of the sensitivity analysis was to assess whether the base case results were sensitive to potential for hidden bias in those treated with delay.

##### DrotAA drug cost

It was assumed that patients who did not receive full infusion of DrotAA and were discharged alive from critical care received 75% (72 hours) of the full DrotAA infusion.

##### Duration of excess mortality

The period of excess mortality was extended beyond the 4 years applied in the base case. Alternative data suggest that, for critical care survivors, excess mortality could continue for up to 25 years [46]. In this sensitivity analysis, the magnitude of excess death rates for an extended period of time (25 years) was taken from a previous study [46] and applied to patients who survived the initial acute hospital episode and all readmissions to the same critical care unit. These excess death rates relative to age- and gender-matched mortality in the general population were applied beyond 4 years for up to 25 years.

##### Health-related quality of life decrement

The HRQOL decrement was assumed to be maintained up to 25 rather than 4 years [46].

##### Health-related quality of life of critical care survivors

The HRQOL of critical care survivors was varied 70% to 90% to that of the general population rather than 80% assumed in the base case.

## Results

### Covariate balance

Before matching, DrotAA patients were, on average, younger (standardized difference of 29%) and had a higher acute physiology score (standardized difference of 50%) and a higher baseline probability of acute hospital death (standardized difference of 30%) in comparison with controls (Table 1). After both Pscore matching and GenMatch, the baseline characteristics were similar between the treatment groups, but the standardized differences were generally lower following GenMatch (Table 1). For the subgroups of patients with two (Table 2a) and three to five (Table 2b) organ systems failing, patient characteristics were well balanced after GenMatch; additional covariates were also evenly distributed between the treatment groups (Additional data file 1). As GenMatch achieved better balance than Pscore matching, further results are reported for GenMatch only.

**Table 1:** Patient characteristics before and after propensity score matching and Genetic Matching

	Mean/percentage^a^ DrotAA (*n *= 1,076)	Mean/percentage^a^ Control (*n *= 1,650)	Percent standardized difference
Age (year)s			
Unmatched	58.70	64.35	28.85
Pscore match	61.48	61.90	2.55
GenMatch	62.22	62.23	0.06
ICNARC model acute physiology score			
Unmatched	30.38	25.09	50.16
Pscore match	27.24	27.18	0.63
GenMatch	27.15	27.15	0.08
Serious conditions in medical history (percentage)			
Cardiovascular			
Unmatched	0.47	1.82	11.63
Pscore match	1.05	1.39	3.09
GenMatch	1.28	1.28	0.00
Respiratory			
Unmatched	2.14	3.39	6.50
Pscore match	2.71	3.02	1.85
GenMatch	1.72	2.64	2.22
Renal			
Unmatched	1.12	2.36	8.31
Pscore match	1.10	1.87	6.33
GenMatch	1.14	1.94	2.32
Liver			
Unmatched	0.84	1.58	5.84
Pscore match	5.53	1.52	21.88
GenMatch	1.14	1.28	0.47
Immunosuppressed			
Unmatched	7.25	10.55	9.75
Pscore match	11.35	9.80	5.06
GenMatch	5.61	8.58	4.10
ICNARC model predicted probability of acute hospital mortality			
Unmatched	0.60	0.51	30.31
Pscore match	0.55	0.55	0.21
GenMatch	0.55	0.55	0.06
Number of organ systems failing during first 24 hours of stay in critical care (percentage)			
Two			
Unmatched	18.40	38.18	38.20
Pscore match	32.15	30.27	4.05
GenMatch	29.35	29.79	0.34
Three			
Unmatched	40.06	38.91	1.91
Pscore match	37.47	41.22	7.69
GenMatch	38.11	41.16	2.20
Four			
Unmatched	33.55	18.12	28.31
Pscore match	25.33	21.48	9.10
GenMatch	26.85	22.71	3.40
Five			
Unmatched	7.99	4.79	10.33
Pscore match	5.05	7.03	8.30
GenMatch	5.69	6.35	0.98
Organ systems failing during first 24 hours of stay in critical care^b ^(percentage)			
Cardiovascular/Respiratory			
Unmatched	14.03	25.27	24.24
Pscore match	23.38	20.74	6.37
GenMatch	23.51	20.73	2.38
Cardiovascular/Respiratory/Acidosis			
Unmatched	33.27	28.49	8.41
Pscore match	28.49	32.32	8.33
GenMatch	32.06	32.80	0.55
Cardiovascular/Respiratory/Renal/Acidosis			
Unmatched	25.37	12.30	26.50
Pscore match	18.50	15.40	8.25
GenMatch	19.15	16.25	2.69
Mechanical ventilation on admission or during first 24 hours of stay in critical care (percentage)			
Unmatched	92.47	75.33	42.51
Pscore match	83.32	82.32	0.93
GenMatch	82.25	82.21	0.03

^a ^Mean reported for continuous variable and percentage for categorical variable

^b^For the types of organ failure, results are presented for the most prevalent type for each number of organ failures; results for other types of organ failure are presented in Additional file 1. DrotAA, Drotrecogin alfa (activated); GenMatch, Genetic Matching; ICNARC, Intensive Care National Audit & Research Centre; Pscore, propensity score.

**Table 2:** Patient characteristics before and after Genetic Matching for each subgroup

	Mean/percentage^a^ DrotAA	Mean/percentage^a^ Control	Percent standardized difference
**a. Subgroup with two organ systems failing**	**(*n *= 198)**	**(*n *= 630)**	
Age (year)s			
Unmatched	57.58	63.04	26.49
GenMatch	61.76	61.82	0.37
ICNARC model acute physiology score			
Unmatched	22.83	20.44	29.53
GenMatch	20.86	20.88	0.35
Serious conditions in medical history, percentage			
Cardiovascular			
Unmatched	1.01	1.75	5.40
GenMatch	1.57	1.57	0.00
Respiratory			
Unmatched	4.55	4.13	1.67
GenMatch	2.78	3.87	2.15
Renal			
Unmatched	2.02	3.81	9.17
GenMatch	1.09	3.14	5.06
Liver			
Unmatched	1.52	1.27	1.69
GenMatch	1.33	1.33	0.00
Immunosuppressed			
Unmatched	7.07	6.98	0.28
GenMatch	4.59	6.16	2.46
ICNARC model predicted probability of acute hospital mortality			
Unmatched	0.42	0.39	10.76
GenMatch	0.39	0.39	0.37
Organ systems failing in first 24 hours^b ^(percentage) - Cardiovascular/Respiratory			
Unmatched	76.26	66.19	18.61
GenMatch	75.60	69.69	4.70
Mechanical ventilation (percentage)			
Unmatched	88.38	70.16	40.02
GenMatch	75.24	75.12	0.10
**b. Subgroup with three to five organ systems failing**	**(*n *= 878)**	**(*n *= 1,020)**	
Age (years)			
Unmatched	58.96	65.16	32.32
GenMatch	62.46	62.47	0.00
ICNARC model acute physiology score			
Unmatched	32.08	27.96	40.83
GenMatch	29.84	29.84	0.01
Serious conditions in medical history (percentage)			
Cardiovascular			
Unmatched	0.34	1.86	13.58
GenMatch	0.58	1.11	2.04
Respiratory			
Unmatched	1.60	2.94	7.78
GenMatch	1.42	2.21	2.09
Renal			
Unmatched	0.91	1.47	4.39
GenMatch	0.90	1.21	1.10
Liver			
Unmatched	0.68	1.77	8.70
GenMatch	1.16	1.16	0.00
Immunosuppressed			
Unmatched	7.29	12.75	15.55
GenMatch	6.11	9.80	4.83
ICNARC model predicted probability of acute hospital mortality			
Unmatched	0.64	0.58	20.12
GenMatch	0.61	0.61	0.00
Number of organ systems failing during first 24 hours of stay in critical care (percentage)			
Three			
Unmatched	49.09	62.94	22.88
GenMatch	54.69	56.74	1.46
Four			
Unmatched	41.12	29.31	20.07
GenMatch	36.57	34.14	1.79
Five			
Unmatched	9.80	7.75	5.82
GenMatch	8.75	9.12	0.46
Organ systems failing during first 24 hours of stay in critical care^b ^(percentage)			
Cardiovascular/Respiratory/Acidosis			
Unmatched	40.77	46.08	8.77
GenMatch	44.10	43.99	0.08
Cardiovascular/Respiratory/Renal/Acidosis			
Unmatched	31.09	19.90	20.64
GenMatch	25.76	24.97	0.64
Mechanical ventilation on admission or during first 24 hours of stay in critical care (percentage)			
Unmatched	93.39	78.53	38.90
GenMatch	85.51	85.51	0.00

^a ^Mean reported for continuous variable and percentage for categorical variable.

^b^For the types of organ failure, results are presented for the most prevalent type for each number of organ failures; results for other types of organ failure are presented in Additional file 1. DrotAA, Drotrecogin alfa (activated); GenMatch, Genetic Matching; ICNARC, Intensive Care National Audit & Research Centre.

### Outcomes - mortality

Table 3 reports odds ratios for the effect of DrotAA on acute hospital mortality within the initial admission. For the overall sample, the odds ratios of death for DrotAA versus control are less than 1 both before and after matching. For the subgroup with two organ systems failing, DrotAA was associated with increased hospital mortality, and the odds ratio after matching was 1.56 (95% CI of 1.25 to 1.85). For the subgroup with three or more organ systems failing, DrotAA was associated with reduced hospital mortality (odds ratio following matching of 0.61, 95% CI of 0.53 to 0.79). The matching has balanced those potential confounders that are observed; for the subgroup with two organ systems failing, the result is that DrotAA is associated with an increase in hospital mortality in excess of 10%.

**Table 3:** Acute hospital mortality and odds ratios for acute hospital mortality for all patients and for each subgroup before and after Genetic Matching

	DrotAA n/N (percentage)	Control n/N (percentage)	Odds ratio (95% CI) for acute hospital mortality for DrotAA versus control
Overall: two to five organ systems failing			
Unmatched	490/1,076 (45.54)	817/1,650 (49.52)	0.85 (0.72, 0.98)
GenMatch	508/1,076 (47.25)	875/1,650 (53.04)	0.79 (0.71, 0.87)
Two organ systems failing			
Unmatched	80/198 (40.40)	221/630 (35.08)	1.26 (0.81, 1.67)
GenMatch	93/198 (46.98)	228/630 (36.23)	1.56 (1.25, 1.85)
Three to five organ systems failing			
Unmatched	410/878 (46.70)	596/1,020 (58.43)	0.62 (0.51, 0.74)
GenMatch	423/878 (48.16)	616/1,020 (60.38)	0.61 (0.53, 0.69)

CI, confidence interval; DrotAA, Drotrecogin alfa (activated); GenMatch, Genetic Matching.

The mean total costs of the initial hospital admission episode were about £32,000 and £15,000 for the DrotAA and control groups (Table 4). The additional costs for DrotAA comprised drugs costs but also additional days in critical care (mean of 15 versus 7.5 days for controls) (Additional data file 2). These additional days in critical care for DrotAA were observed for both survivors (mean of 17 versus 9 days) and decedents (mean of 11 versus 5 days). Approximately 10% of both DrotAA patients and controls were readmitted over the course of 4 years (Additional data file 3); hence, readmission costs were similar across the groups. Similarly, for both subgroups, DrotAA was associated with higher costs during the initial acute hospital stay (Table 4).

**Table 4:** Costs, in pounds sterling, of initial hospital episode and readmissions within 4 years

	DrotAA, mean (SD)	Control, mean (SD)
Overall: two to five organ systems failing		
Drug cost	5,022 (1,688)	0 (0)
ICU costs	21,204 (19,660)	10,692 (13,533)
Hospital costs	5,812 (9,145)	4,411 (7,884)
Total initial episode costs	32,038 (24,285)	15,102 (16,787)
ICU readmission costs	1,729 (8,252)	1,276 (5,987)
Hospital costs	2,281 (14,694)	2,054 (11,646)
Total readmission costs	4,010 (21,380)	3,330 (16,057)
Two organ systems failing		
Drug cost	5,328 (1,338)	0 (0)
ICU costs	17,364 (15,564)	8,806 (9,142)
Hospital costs	5,933 (8,889)	4,998 (7,168)
Total initial episode costs	28,625 (19,810)	13,804 (12,108)
ICU readmission costs	914 (3,941)	916 (3,894)
Hospital costs	1,652 (5,225)	1,667 (8,538)
Total readmission costs	2,567 (8,202)	2,684 (11,308)
Three to five organ systems failing		
Drug cost	4,916 (1,773)	0 (0)
ICU costs	22,853 (20,504)	11,692 (14,921)
Hospital costs	5,776 (9,496)	3,967 (7,898)
Total initial episode costs	33,544 (25,342)	15,659 (18,311)
ICU readmission costs	2,152 (9,978)	1,120 (5,604)
Hospital costs	2,823 (20,799)	1,794 (11,361)
Total readmission costs	4,976 (28,531)	2,914 (15,488)

Overall (two to five organ systems failing): total number = 2,726, Drotrecogin alfa (activated) (DrotAA) = 1,076, control = 1,650; two organ systems failing: total number = 828, DrotAA = 198, control = 630; three to five organ systems failing: total number = 1,898, DrotAA = 878, control = 1,020. ICU, intensive care unit.

### Lifetime cost-effectiveness results

**Figure 1:**
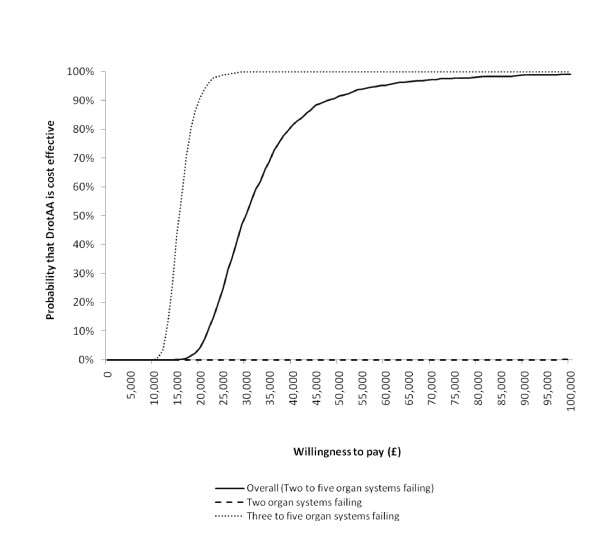
**Cost-effectiveness acceptability curves**. The curves show the probability that the intervention is cost-effective at different levels of willingness to pay for a quality-adjusted life year gain. DrotAA, Drotrecogin alfa (activated).

The lifetime incremental cost-effectiveness results are reported in Table 5. For the overall sample, the incremental costs for DrotAA were £18,000 and the QALY gain was 0.60, leading to an ICER of £30,158 per QALY. The QALY gain of 0.6 valued at £20,000 led to an INB of -£6,000 (0.6*20,000-18,000). For patients with two organ systems failing, the higher mortality associated with DrotAA led to an average loss of one QALY, which, coupled with the positive incremental costs, led to a negative INB (-£35,000). For the subgroup with three to five organ systems failing, the incremental QALY for DrotAA was relatively high (mean QALY gain of 1.3), the ICER was relatively low (£15,561), and the corresponding INB was positive (£6,000) (Table 5). The statistical uncertainty around the CEA results is summarized in the cost-effectiveness acceptability curves in Figure 1. The probabilities that DrotAA is cost-effective at a threshold of £20,000 per QALY are 5% for the overall sample, 0% for the subgroup with two organ systems failing, and 99% for the subgroup with three or more organ systems failing.

**Table 5:** Lifetime costs in pounds sterling, quality-adjusted life years, and incremental net benefits in pounds sterling after Genetic Matching

	DrotAA, mean (SD)	Control, mean (SD)	Incremental, mean (95% CI)
Overall: two to five organ systems failing			
Lifetime costs	36,048 (35,522)	18,432 (26,708)	17,616 (15,959 to 19,273)
Lifetime QALYs	5.70 (6.57)	5.11 (6.61)	0.58 (0.24 to 0.93)
INB^a^	77,896 (131,138)	83,830 (129,324)	-5,934 (-12,735 to 868)
Two organ systems failing			
Lifetime costs	31,191 (21,959)	16,488 (18,399)	14,703 (12,763 to 16,644)
Lifetime QALYs	5.87 (6.54)	6.83 (6.75)	-0.97 (-1.62 to -0.32)
INB^a^	86,117 (130,821)	120,148 (134,047)	-34,031 (-47,028 to -21,034)
Three to five organ systems failing			
Lifetime costs	38,520 (43,205)	18,572 (27,266)	19,948 (17,610 to 22,286)
Lifetime QALYs	5.63 (6.59)	4.35 (6.33)	1.28 (0.86 to 1.70)
INB^a^	74,038 (132,559)	68,348 (122,496)	5,690 (-2,543 to 13,924)

Overall (two to five organ systems failing): total number = 2,726, Drotrecogin alfa (activated) (DrotAA) = 1,076, control = 1,650; two organ systems failing: total number = 828, DrotAA = 198, control = 630; three to five organ systems failing: total number = 1,898, DrotAA = 878, control = 1,020. ^a^Incremental net benefits (INBs) are calculated by following method guidance [36] and multiplying the mean quality-adjusted life year (QALY) gain (or loss) by £20,000 and subtracting from this the incremental cost. CI, confidence interval; SD, standard deviation.

### Sensitivity analysis

**Figure 2:**
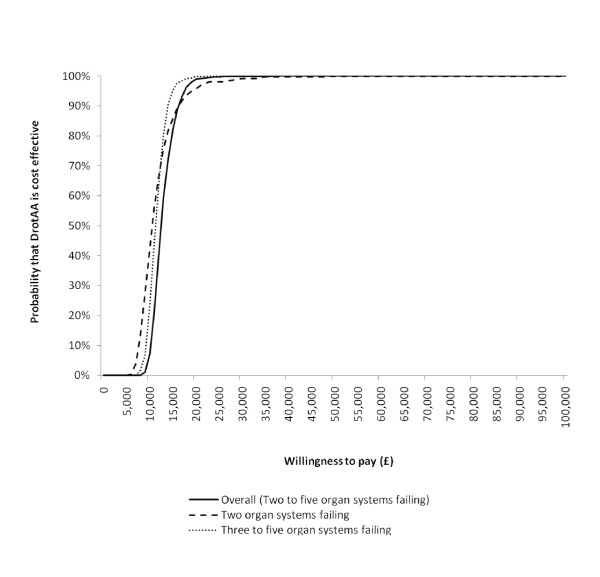
**Cost-effectiveness acceptability curves for DrotAA within 24 hours of critical care admission**. The curves show the probability that the intervention is cost-effective at different levels of willingness to pay for a quality-adjusted life year gain for the subsample who received Drotrecogin alfa (activated) (DrotAA) within 24 hours of admission to the critical care unit.

The impact on the base case results of matching just with the Pscore rather than the individual covariates (GenMatch), of limiting DrotAA to within 24 hours, of increasing the drug cost, and of changing the duration of excess mortality and HRQOL decrement are shown in Table 6. When the sample was restricted to those who received DrotAA during the first 24 hours in critical care, the DrotAA cases were reduced by 36%. For this subsample, acute hospital mortality following DrotAA was reduced, particularly for the subgroup with two organ systems failing (Additional data file 4). Here, the odds ratio for DrotAA versus control was 0.64 (95% CI of 0.50 to 0.78), resulting in a QALY gain of 1.2 and an ICER of £11,000. As the accompanying cost-effectiveness acceptability curve shows, if the sample is limited to those commencing DrotAA within 24 hours, then the probability that the intervention is cost-effective exceeds 0.90 for both subgroups, irrespective of the willingness to pay for a QALY gained (Figure 2). The results were robust to the other main assumptions made in the base case (Table 6).

**Table 6:** Sensitivity analysis on incremental net benefits in pounds sterling

	Overall: two to five organ systems failing	Two organ systems failing	Three to five organ systems failing
Base case	-5,934 (-12,735 to 868)	-34,031 (-47,028 to -21,034)	5,690 (-2,543 to 13,924)
Pscore matching	-7,641 (-13,213, -2,069)	-32,846 (-44,704, -20,987)	391 (-6,350, 7,133)
DrotAA given within 24 hours^a^	8,078 (733 to 15,423)	11,131 (-2,173 to 24,435)	12,387 (3,491 to 21,283)
Cost of drug (alternative assumption)	-4,687 (-11,544 to 2,171)	-33,232 (-46,309 to -20,154)	7,339 (-976 to 15,653)
Excess mortality up to 25 years	-6,240 (-12,897 to 418)	-33,580 (-46,300 to -20,860)	5,046 (-3,012 to 13,104)
Excess reduction in HRQOL up to 25 years	-7,240 (-13,328 to -1,152)	-31,895 (-43,509 to -20,280)	2,863 (-4,512 to 10,237)
HRQOL of critical care survivors is 70% that of general population	-6,029 (-12,679 to 621)	-32,176 (-44,912 to -19,440)	4,839 (-3,202 to 12,880)
HRQOL of critical care survivors is 90% that of general population	-5,347 (-12,254 to 1,560)	-33,134 (-46,353 to -19,915)	6,239 (-2,117 to 14,596)

Values are presented as mean (95% confidence interval). Incremental net benefits are calculated by multiplying the mean quality-adjusted life year gain (or loss) by £20,000 and subtracting from this the incremental cost. ^a^Overall (two to five organ systems failing): total number = 2,337, Drotrecogin alfa (activated) (DrotAA) = 687, control = 1,650; two organ systems failing: total number = 727, DrotAA = 97, control = 630; three to five organ systems failing: total number = 1,610, DrotAA = 590, control = 1,020. HRQOL, health-related quality of life; Pscore, propensity score.

## Discussion

This study reports the cost-effectiveness of DrotAA in routine clinical practice for adult severe sepsis patients admitted to critical care units in England, Wales, and Northern Ireland. The results suggest that DrotAA is cost-effective for patients at high risk of acute hospital death (three to five organ systems failing during the first 24 hours in critical care). For patients with two organ systems failing, the results are less clear; whereas the base case finding suggested that DrotAA was not cost-effective, the sensitivity analysis suggested that the intervention was cost-effective if limited to those patients who received DrotAA within 24 hours.

This study extends previous CEAs of DrotAA by using data from a large sample of units delivering routine clinical care; previous CEAs used PROWESS data [18-23,62]. Our approach allows cost-effectiveness results to be presented for patients with characteristics relevant to a more general population with severe sepsis. We also report CEA results for patient subgroups defined according to the number of organ systems failing, reflecting different baseline risks of death. There is no consensus on how best to define risk for patients with severe sepsis; some recommendations, notably in the US, favor the use of APACHE II score [63], whereas others prefer the number of organ systems failing [29,41]. More generally, there is a clear consensus that, as in this study, subgroups should be defined *a priori *[41]. This study also extends previous CEAs of DrotAA by capturing important aspects of the longer-term impact of DrotAA in that readmissions and accompanying mortality are recorded for 4 years. This provides a more robust basis for extrapolating long-term outcomes than previous approaches [18,19,21].

A further strength of this study is that it uses appropriate methods to address selection bias by applying both Pscore matching and GenMatch, which are recommended approaches for addressing baseline differences between treatment groups in potential confounding factors. Both Pscore and GenMatch achieved good balance on key prognostic factors, and GenMatch achieved excellent balance both overall and for each subgroup. Hence, the possibility of bias arising from differences in observed factors was minimized.

Short-term mortality estimates of this study are broadly consistent with those from PROWESS. We found that DrotAA reduced absolute acute hospital mortality by 5.8% in comparison with a 6.1% reduction in 28-day mortality in the PROWESS study. Our odds ratios (0.79, 95% CI 0.71 to 0.87) are slightly less favorable for DrotAA than in PROWESS (0.8, 95% CI 0.69 to 0.94). The somewhat smaller effect of DrotAA on mortality leads to lower lifetime QALY gains compared with those of previous studies based on PROWESS [18,19,21,22,62]. Our lifetime incremental costs of DrotAA (£18,000) exceeded previous estimates (less than £6,000) [19,21,62], which reflected the relatively high initial LOS for DrotAA patients managed in routine clinical practice (mean of 15 days for DrotAA versus 7.5 days for the control group). Consequently, our estimates of the long-term cost-effectiveness of DrotAA are less favorable than those previously reported [18,22]. Dhainaut and colleagues [64], applying Pscore matching, also observed less favorable cost-effectiveness ratios; the authors reported a cost per QALY of €34,000 in routine clinical practice in France.

When methodological guidelines are followed and cost-effectiveness results are reported over the lifetime, assumptions inevitably have to be made. Our sensitivity analysis suggested that the findings, with one exception, were generally insensitive to the assumptions made. The base case results reported that DrotAA was not cost-effective for the subgroup with two organ systems failing. If the sample is limited to those cases receiving DrotAA administered early (within 24 hours of admission), then the intervention, on average, reduces mortality for the subgroup with two organ systems failing and becomes cost-effective. Several other studies have indicated the benefit of early treatment. The ENHANCE (Extended Evaluation of Recombinant Human Activated Protein C) trial showed that treatment within 24 hours of organ failure with DrotAA was associated with 23% lower odds of death at 28 days in comparison with treatment more than 24 hours after sepsis-induced organ failure [25]. Others also have suggested that DrotAA is more effective when administered early [24,29].

This study has several limitations. Firstly, we assumed that acute hospital survivors who were not readmitted to critical care faced a death rate equal to that of the general population (age- and gender-matched), and this may have underestimated deaths in this specific group of patients. Our estimated death rates were similar to those used previously; for example, our overall projected mortality at 5-year follow-up was 49% versus 47% in Wright and colleagues [40]. Furthermore, the sensitivity analysis finds that the CEA results are insensitive to this assumption. Secondly, like other investigators attempting to report lifetime cost-effectiveness, we had to make plausible assumptions about the long-term HRQOL; we tested these assumptions in the sensitivity analysis and found that they had little impact on the results. Thirdly, unbiased treatment effects from matching are based on the assumption of no hidden bias; this assumption might be less tenable for the subgroup with two organ systems failing, in which 36% of the DrotAA cases are treated after a delay of more than 24 hours. There is some limited evidence from the FDA *post hoc *analysis [15] and ADDRESS trial [12] to suggest that DrotAA may increase mortality in subgroups with low baseline risk. However, our sensitivity analysis suggested that, if the sample was limited to those treated within 24 hours, DrotAA was more effective and was cost-effective for the subgroup with two organ systems failing. Here, the case severity at the time DrotAA infusion commenced might have been worse than that measured at baseline and used in the matching. This suggests that either the base case findings for the subgroup with two organ systems failing is prone to hidden bias or DrotAA is more effective and cost-effective for this subgroup if administered early. Further research is required to focus specifically on the effectiveness and cost-effectiveness of DrotAA administration for patients at low levels of baseline severity and according to the timing of therapy initiation.

## Conclusions

This CEA, based on a prospective cohort study, suggests that, in routine clinical practice, DrotAA is cost-effective for severe sepsis patients with three or more organ systems failing within 24 hours of admission to a critical care unit. For patients with two organ systems failing, this study could not provide unequivocal evidence on the cost-effectiveness of DrotAA.

## Key messages

• Drotrecogin alfa (activated), or DrotAA, is cost-effective in routine practice for severe sepsis patients with three to five organ systems failing during the first 24 hours in critical care.

• For patients with two organ systems failing, it is unclear whether DrotAA is cost-effective.

• Further research is required on the effectiveness and cost-effectiveness of DrotAA administration for patients at low levels of baseline severity and according to the timing of therapy initiation.

## Abbreviations

ADDRESS: Administration of Drotrecogin alfa (activated) in Early Stage Severe Sepsis; APACHE II: Acute Physiology and Chronic Health Evaluation II; CEA: cost-effectiveness analysis; CI: confidence interval; CMP: Case Mix Programme; DrotAA: Drotrecogin alfa (activated); EMEA: European Medical Evaluation Agency; FDA: US Food and Drug Administration; GenMatch: Genetic Matching; HRQOL: health-related quality of life; ICER: incremental cost-effectiveness ratio; ICNARC: Intensive Care National Audit & Research Centre; IMscore: Intensive Care National Audit & Research Centre model physiology score; INB: incremental net benefit; LOS: length of stay; PROWESS: Protein C Worldwide Evaluation in Severe Sepsis; Pscore: propensity score; QALY: quality-adjusted life year; RCT: randomized controlled trial.

## Competing interests

ICNARC conducts paid for analyses for industry, including Eli Lilly and Company (Indianapolis, IN, USA). DAH and KMR are employees of ICNARC. BHC has received honoraria for consultancies and speaking from Eli Lilly and Company.

## Authors' contributions

ZS and RG participated fully in the design of this study, the analysis of the data, and the writing of this paper. DAH and KMR participated fully in the design of this study, the collection of data, the analysis of the data, and the writing of this paper. BHC participated fully in the analysis of the data and in the writing of this paper. All authors read and approved the final manuscript.

## Supplementary Material

Additional file 1 **Additional patients' characteristics before and after Pscore matching and GenMatch**. Balance statistics of additional covariates are shown. Click here for file

Additional file 2 **Length of stay (days) in critical care at index admission - mean (sd)**. Shown is the length of stay in critical care in DrotAA and control before and after GenMatch. Click here for file

Additional file 3 **Readmission to original critical care unit within 4 years (n/N(%)) and mortality at readmission (n/N(%))**. Readmissions and observed deaths in four years in original critical care unit are shown. Click here for file

Additional file 4 **Odds ratio, lifetime costs (£), QALYs, incremental net benefits (INB) (£) mean (95% CI) for the subsample who received DrotAA within 24 hours of admission to the critical care unit**. Odds ratio, lifetime costs, QALYs, and incremental net benefits are shown where DrotAA is given within 24 hours of admission. Click here for file
