# Supplementary material for: Is Drotrecogin alfa (activated) for adults with severe sepsis, cost-effective in routine clinical practice?
Source: Crit Care. 2011 Sep 26;15(5):R228. doi: 10.1186/cc10468 (PMC3334774; doi:10.1186/cc10468)
Supplement: Additional file 1 — Additional patients' characteristics before and after Pscore matching and GenMatch. Balance statistics of additional covariates are shown. [file cc10468-S1.DOC]

**Additional file 1: Additional patients’ characteristics before and after Pscore matching and GenMatch**

|  | | **Mean**  **DrotAA** | **Mean**  **Control** | **Percent**  **standardised difference** |
| --- | --- | --- | --- | --- |
| **a) Overall (two to five organ systems failing)^*^** | | **(n=1,076)** | **(n=1,650)** |  |
| **Sex (% of male)** | Unmatched | 50.47 | 54.06 | 5.88 |
|  | Pscore match | 52.18 | 51.44 | 0.52 |
|  | GenMatch | 50.48 | 52.94 | 1.74 |
| **Other categories of organ system failing during first 24 hours of stay in critical care** | |  |  |  |
| Cardio/Acidosis | Unmatched | 0.37 | 2.42 | 16.47 |
|  | Pscore match | 1.45 | 1.60 | 0.43 |
|  | GenMatch | 0.81 | 1.54 | 2.41 |
| Cardio/Respiratory/Renal | Unmatched | 3.07 | 3.76 | 3.16 |
|  | Pscore match | 3.25 | 3.64 | 0.75 |
|  | GenMatch | 2.31 | 3.08 | 1.68 |
| Cardio/Respiratory/Haematological/Acidosis | Unmatched | 7.34 | 4.67 | 8.90 |
|  | Pscore match | 6.16 | 4.99 | 1.80 |
|  | GenMatch | 7.15 | 5.47 | 2.46 |
| **b) Two organ systems failing subgroup^**^** | | **(n=198)** | **(n=630)** |  |
| **Sex (% of male)** | Unmatched | 57.07 | 56.67 | 0.67 |
|  | GenMatch | 52.66 | 56.40 | 2.66 |
| **Other categories of organ system failing during first 24 hours of stay in critical care** | |  |  |  |
| Cardio/Acidosis | Unmatched | 2.02 | 6.35 | 19.45 |
|  | GenMatch | 2.42 | 4.95 | 4.77 |
| **c) Three to five organ systems failing subgroup^**^** | | **(n=878)** | **(n=1,020)** |  |
| **Sex (% of male)** | Unmatched | 48.98 | 52.45 | 5.68 |
|  | GenMatch | 48.37 | 53.06 | 3.32 |
| **Other categories of organ system failing during first 24 hours of stay in critical care** | |  |  |  |
| Cardio/Respiratory/Renal | Unmatched | 3.76 | 6.08 | 9.12 |
|  | GenMatch | 4.16 | 5.01 | 1.43 |
| Cardio/Respiratory/Haematological/Acidosis | Unmatched | 9.00 | 7.55 | 4.24 |
|  | GenMatch | 9.80 | 7.85 | 2.43 |

^*^ Overall (two to five organ systems failing) patient characteristics are reported before and after Pscore matching and GenMatch.

^**^ Patient characteristics for subgroups are reported before and after GenMatch.
