# Supplementary material for: Is Drotrecogin alfa (activated) for adults with severe sepsis, cost-effective in routine clinical practice?
Source: Crit Care. 2011 Sep 26;15(5):R228. doi: 10.1186/cc10468 (PMC3334774; doi:10.1186/cc10468)
Supplement: Additional file 2 — Length of stay (days) in critical care at index admission - mean (sd). Shown is the length of stay in critical care in DrotAA and control before and after GenMatch. [file cc10468-S2.DOC]

Additional file 2: Length of stay (days) in critical care at index admission – mean (sd)

|  |  | **DrotAA** | **Control** |
| --- | --- | --- | --- |
| Overall (two to five organ systems failing) | Unmatched | 15.23  (13.89) | 7.09  (8.47) |
|  | GenMatch | 14.88  (13.48) | 7.56  (9.26) |
| Two organ systems failing | Unmatched | 15.39  (14.41) | 6.76  (7.12) |
|  | GenMatch | 14.47  (12.97) | 7.34  (7.62) |
| Three to five organ systems failing | Unmatched | 15.20  (13.78) | 7.29  (9.20) |
|  | GenMatch | 15.03  (13.26) | 7.75  (9.85) |
