# Supplementary material for: Is Drotrecogin alfa (activated) for adults with severe sepsis, cost-effective in routine clinical practice?
Source: Crit Care. 2011 Sep 26;15(5):R228. doi: 10.1186/cc10468 (PMC3334774; doi:10.1186/cc10468)
Supplement: Additional file 3 — Readmission to original critical care unit within 4 years (n/N(%)) and mortality at readmission (n/N(%)). Readmissions and observed deaths in four years in original critical care unit are shown. [file cc10468-S3.DOC]

Additional file 3: Readmission to original critical care unit within 4 years (n/N(%)) and mortality at readmission (n/N(%))

|  |  | **DrotAA** | **Control** |
| --- | --- | --- | --- |
| Readmissions (% of total) | Overall (two to five organ systems failing) | 128/1,076  11.90 | 180/1,650  10.91 |
|  | Two organ systems failing | 26/198  13.13 | 79/630  12.54 |
|  | Three to five organ systems failing | 102/878  11.62 | 101/1,020  9.90 |
| Deaths in hospital following readmission n (%) | Overall (two to five organ systems failing) | 43/1,076  4.00 | 70/1,650  4.24 |
|  | Two organ systems failing | 5/198  2.53 | 29/630  4.60 |
|  | Three to five organ systems failing | 38/878  4.33 | 41/1,020  4.02 |

* Overall (two to five organ systems failing): N=2,726, DrotAA =1,076, control =1,650;

Two organ systems failing: N= 828, DrotAA =198, control =630;

Three to five organ systems failing: N= 1,898, DrotAA =878, control =1,020
