# Supplementary material for: Is Drotrecogin alfa (activated) for adults with severe sepsis, cost-effective in routine clinical practice?
Source: Crit Care. 2011 Sep 26;15(5):R228. doi: 10.1186/cc10468 (PMC3334774; doi:10.1186/cc10468)
Supplement: Additional file 4 — Odds ratio, lifetime costs (£), QALYs, incremental net benefits (INB) (£) mean (95% CI) for the subsample who received DrotAA within 24 hours of admission to the critical care unit. Odds ratio, lifetime costs, QALYs, and incremental net benefits are shown where DrotAA is given within 24 hours of admission. [file cc10468-S4.DOC]

Additional file 4: Odds ratio, lifetime costs (£), QALYs, incremental net benefits (INB) (£) mean (95% CI) for the subsample who received DrotAA within 24 hours of admission to the critical care unit

|  | **Overall (two to five organ systems failing)** | **Two organ systems failing** | **Three to five organ systems failing** |
| --- | --- | --- | --- |
| Odds ratio | 0.65  (0.57 to 0.72) | 0.64  (0.50 to 0.78) | 0.58  (0.49 to 0.66) |
| Incremental cost | 13,645  (12,061 to 15,230 ) | 13,144  (11,175 to 15,113) | 15,896  (13,887 to 17,904) |
| Incremental QALY | 1.09  (0.72 to 1.46) | 1.21  (0.55 to 1.88) | 1.41  (0.96 to 1.87) |
| INB^$^ | 8,078  (733 to 15,423) | 11,131  (-2,173 to 24,435) | 12,387  (3,491 to 21,283) |

* Overall (two to five organ systems failing): N=2,337, DrotAA =687, control =1,650;

Two organ systems failing: N= 727, DrotAA =97, control =630;

Three to five organ systems failing: N= 1,610, DrotAA =590, control =1,020

^$^ QALY gains (or losses) are valued at £20,000 in INB calculation.
